# Arsenic

**Published:** 1865-11

**Authors:** 


### ARSENIC.

The *Abeille Medicale* contains an interesting article on the medical appliances of this deadly poison. The author, Dr. Papillion, of Saujon, Charante-Inferieure, maintains, in allusion to a paper by Dr. Wahn, addressed to the Academy of Medicine and advocating the same views, that arsenic, instead of being a debilitating drug, is on the contrary a strengthener, and one of the best remidies in case of cachexy in marshy districts, king's evil, lymphatism, chlorosis, and even consumption.

Against this opinion there is an experiment of Dr. Briquet's, which consists in measuring the pressure of arterial blood after an arsenical injection into the veins. As the pressure appears to diminish, Dr. Briquet infers therefrom that arsenic exercises a depressing influence on the animal economy. But to this our author replies that any drug whatever, even iron, the greatest strengthener known, will cause weakness, and even death when so misapplied against the laws of nature. On the other hand, he contends that he has seen pale, sallow, weak, and thin patients use iron, vegetable tonics, and cod liver oil for months and years without the slightest benefit; and those same patients acquire appetites, strength, flesh, and a healthy complexion on taking daily doses of a few milligrams of arsenic. He denies that arsenic may momentarily act as a stimulant, though when absorbed it ultimately debilitates: and declares that he has caused arsenic to be taken daily for years together, and always found it induce regularity in the nutritive functions.

He adds that he has himself, for the last five or six years, been taking a daily dose of two milligrams of arseniate of antimony, and that to this practice he not only attributes the cessation of palpitations of the heart with which he was afflicted, and which prevented his sleeping on his left side, but also a considerable improvement in his general health, and the disappearance of violent headaches which he used to have once a week for twenty-four hours at a time. Dr. Devergie, he further observes, has admitted the tonic qualities of arsenic, but only in the beginning : a continual use of this remedy, according to this practitioner, induces debility. To this Dr. Papillion replies that arsenic is generally administered under the form of Fowler's drops, or of Asiatic pills, with variable proportions of arsenious acid, the doses increasing progressively. In this way the body gradually receives more arsenic than is necessary, and it then of course acts like every other tonic, which by excess loses its strengthening power, and becomes debilitating ; but the dose of two milligrams may be continued with impunity for any number of years without any other effect but that of stimulating and renovating the vital powers. — *The Medical and Surgical Reporter.*

